# Key Insights into Hand Biomechanics: Human Grip Stiffness Can Be Decoupled from Force by Cocontraction and Predicted from Electromyography

**DOI:** 10.3389/fnbot.2017.00017

**Published:** 2017-05-22

**Authors:** Hannes Höppner, Maximilian Große-Dunker, Georg Stillfried, Justin Bayer, Patrick van der Smagt

**Affiliations:** ^1^Bionics Lab, Institute of Robotics and Mechatronics, German Aerospace Center DLR e.V., Oberpfaffenhofen, Wessling, Germany; ^2^Department of Informatics, Technische Universität München, Munich, Germany; ^3^fortiss, TUM affiliated Institute, Munich, Germany

**Keywords:** grip stiffness, cocontraction, grip force, intrinsic hand muscles, interosseus muscles, electromyography, soft robotics, variable-stiffness actuators

## Abstract

We investigate the relation between grip force and grip stiffness for the human hand with and without voluntary cocontraction. Apart from gaining biomechanical insight, this issue is particularly relevant for variable-stiffness robotic systems, which can independently control the two parameters, but for which no clear methods exist to design or efficiently exploit them. Subjects were asked in one task to produce different levels of force, and stiffness was measured. As expected, this task reveals a linear coupling between force and stiffness. In a second task, subjects were then asked to additionally decouple stiffness from force at these force levels by using cocontraction. We measured the electromyogram from relevant groups of muscles and analyzed the possibility to predict stiffness and force. Optical tracking was used for avoiding wrist movements. We found that subjects were able to decouple grip stiffness from force when using cocontraction on average by about 20% of the maximum measured stiffness over all force levels, while this ability increased with the applied force. This result contradicts the force–stiffness behavior of most variable-stiffness actuators. Moreover, we found the thumb to be on average twice as stiff as the index finger and discovered that intrinsic hand muscles predominate our prediction of stiffness, but not of force. EMG activity and grip force allowed to explain 72 ± 12% of the measured variance in stiffness by simple linear regression, while only 33 ± 18% variance in force. Conclusively the high signal-to-noise ratio and the high correlation to stiffness of these muscles allow for a robust and reliable regression of stiffness, which can be used to continuously teleoperate compliance of modern robotic hands.

## 1 Introduction

Stiffness is an important property for the interaction of any biological or mechanical system with its environment. A soft system (low stiffness) will yield to external perturbation forces, while a stiff system will withstand them. For example, when brushing one’s teeth, the grip on the toothbrush needs to be soft enough for following the shape of the jaw without hurting the gum, but firm enough (high stiffness) for keeping the handle within a stable pose without losing it and for guiding the head of the toothbrush in the desired direction.

Stiffness is defined as a ratio of a force change to a corresponding displacement. However, additional criteria need to be fulfilled for a force–displacement relation to be considered stiffness (Latash and Zatsiorsky, 1993). These criteria are resistance, passivity, and elasticity: the direction of the force change opposes the direction of the displacement (resistance of the system against deformation); for the force change, no external energy is supplied (passivity); the force change is only dependent on the displacement and has a conservative nature (elasticity). The elasticity criterion also ensures that the reaction is instantaneous, since otherwise, the force change would not only depend on the displacement but also on the time.

A resistive response to perturbations can also be provided by the human body via reflexes, which are involuntary contractions of muscles that involve the travel of nervous signals from sensory receptors via the central nervous system to the muscles. Despite being sometimes called “reflexive stiffness,” this kind of response falls outside of our definition of stiffness, because the contraction of the muscle consumes energy and the travel of the nervous signal introduces a delay. Our definition of stiffness also excludes force changes due to acceleration (inertial forces) and velocity (damping forces). Conclusively, the stiffness we measure is not a quasi-stiffness, reflexive stiffness, nor apparent stiffness [see also Latash and Zatsiorsky (1993)].

In biomechanics and neuroscience, our definition of stiffness is commonly referred to by using the terms *static*, *intrinsic*, or *a-reflexive* stiffness and is close to the stiffness of mechanical springs. It is a combination of passive stiffness stemming from the muscles, tendons, surrounding tissue, and ligaments and short-range stiffness originating from the crossbridges.

It has been shown that (a) the stiffness of a muscle increases linearly with increasing muscle force (Zajac, 1989; Shadmehr and Arbib, 1992) and the stiffness of a grip increases linearly with grip force (Höppner et al., 2011; Van Doren, 1998); (b) the slope of the linear force–stiffness curve can be modulated by changing the posture of the limb (kinematics) (Höppner et al., 2013); and (c) by simultaneously contracting flexor and extensor muscles (cocontraction), stiffness can be varied without changing posture when no force is applied to the environment (zero net force) (Osu et al., 2002). In this article, we investigate the open question whether (d) cocontraction can be used to decouple stiffness from its linear increase with force while external forces are applied and kinematics are kept constant.

Each of the stiffness modulation methods has different advantages: while changing kinematics is energy efficient, external force modulation and cocontraction allow for posture maintenance. Among these methods, we choose to investigate cocontraction as stiffness modulation mechanism, because it raises open biomechanical questions and its results can be directly applied to variable-stiffness actuators in robots. By using a perturbation device that can measure human grip stiffness related to grip force (Höppner et al., 2011, 2013), we can investigate the human mechanism of cocontraction. The device is able to measure an almost exact representation of pure stiffness—which is captured by the terms passivity, resistance, and elasticity—imposing that it is able to refrain from measuring influences from active feedback or damping and inertia. For this, the device measures forces at two static positions (see Figure 5), so that the acceleration and velocity are zero during the measurements, and accomplishes the transition between the two positions fast enough to exclude the possibility of reflexes. Furthermore, we use EMG—since it possibly allows measuring muscle states continuously and is thus highly relevant for teleoperation in robotics—to investigate the possibility to regress force and stiffness from the measurement of muscular activity from relevant intrinsic and extrinsic hand muscles. Note that unlike with reflexes, the metabolic energy cost for maintaining the static muscle tension does not affect the passivity criterion, because it is only used to establish the state of the system prior to the perturbation and is not affected by the displacement-related force change.

### 1.1 Stiffness in Robots

Actively controlled compliant robotic systems (Albu-Schäffer and Hirzinger, 2002) are able to mimic an *apparent stiffness*, which makes them suitable for human–robot interaction. However, similar to the human reflex, they reach their limits at high-frequency impacts (Hogan, 1984). Thus, these systems have been extended recently by further adding an intrinsic elasticity (Vanderborght et al., 2013; Grebenstein, 2014; Wolf et al., 2016) by the use of non-linear springs—variable-stiffness actuators (VSA)—which is a concept copied from the flexibility found in biological limbs: through *cocontraction*, we can increase the stiffness and damping characteristics of our limbs, thus influencing the energy exchange characteristics with our environment. Besides (a) allowing to compensate high-frequency impacts and increasing system robustness, VSAs offer valuable properties such as (b) enriching dynamic capabilities by allowing to frequently store energy in reversal points or (c) embodying the desired behavior of a task into the mechanical structure of the robot (Visser et al., 2011). One of their main characterizing properties is their torque–stiffness diagram (Wolf et al., 2015, 2016), showing the basic coupling between torque and stiffness and how it varies with *pretensioning* of the joint—which is similar to the mechanism of cocontraction found in humans. However, biomechanics is essentially lacking similar diagrams for the human locomotor system, which might be used by robotic engineers as a template. Hence, heuristic methods have been used for setting properties of VSAs rather than clear design guidelines; e.g., most of the VSAs have a rather limited performance in decoupling stiffness from torque for the higher torques. This article is trying to close this gap in biomechanics and to find an answer to the main question: Can stiffness be significantly decoupled from its linear increase with force with cocontraction during posture maintenance?

### 1.2 Cocontraction

**Figure 1:**
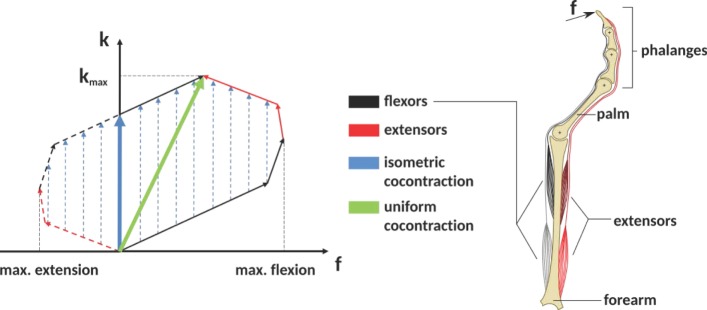
**The expected influence of cocontraction on the Cartesian net force *f* and stiffness *k* at the fingertip (exemplary)**. The theoretically achievable force–stiffness range of an antagonistic system consisting of a set of joints actuated by two flexor and two extensor muscles (assumption: linear dependence between force and stiffness of a single muscle). The field is defined by the vector sum of the single muscle curves. Uniform cocontraction leads to an increase in force and stiffness along a line pointing in the direction of maximum stiffness *(green)*, and isometric cocontraction leads to an increase in stiffness, but not in Cartesian net force *(blue)*.

Cocontraction is the simultaneous activation of at least two antagonistic muscles acting on a joint (Gribble et al., 2003). See Figure 1 as an example of how cocontraction of antagonistic muscles affects the force and stiffness measured at an end-effector: it depicts a diagram of the force and stiffness at the fingertip of a simplified finger actuated by two flexor and two extensor muscles. The red and black arrows denote the linear force–stiffness relations of single a-reflexive muscles with the arrow’s tips pointing to the muscle’s maximum force and stiffness. Although activating flexor (black arrows) and extensor (red arrows) muscles will contribute to stiffness in a positive way, the flexor muscle activation will increase the applied force and extensor muscle activation will decrease the applied force. Assuming a linear relation between force and stiffness, the reachable force–stiffness range of an antagonistic setup is defined by the vector sum of the single force–stiffness relations of the single antagonistic muscles [similar to the quadrilateral region of two antagonist muscles defined in the study by Kearney and Hunter (1990)]. If humans were able to activate all muscles independently, they would be able to reach the entire area by cocontraction. However, it is well known that due to neural and mechanical synergies, they are not able to independently activate them (De Luca and Mambrito, 1987; Milner, 2002).

In literature, it remains unclear what the notion cocontraction exactly means. Sometimes it refers to a *uniform* scaling of all muscular activations between their minimum and maximum values, resulting in an increase of force and stiffness along the direction pointing to the maximum stiffness (green arrow in Figure 1). Contrarily, an *isometric* cocontraction will increase stiffness only and keep the applied force constant (blue arrows)—similar to the notion *pretension* used for VSAs in robotics. Since we focus on robotics, we will ask subjects for an isometric cocontraction only and will give them a visual feedback about the applied force and stiffness. Furthermore, by referring to the notation cocontraction, we mean the simultaneous contraction of flexor and extensor muscles of thumb and index finger, which results in stable pinch grip force but increased pinch grip stiffness. A simultaneous contraction of all flexor muscles of thumb and index finger opposing each other in a pinch is not considered as cocontraction in this article.

Moreover, by referring to the notation *decoupling*, we naturally imply an increase of stiffness from its usual coupling to force. We will refrain from analyzing the possibility to decrease stiffness from its normal coupling to force—since it is expected to be impossible.

Different studies simulated, measured, and analyzed the role of cocontraction for the human locomotor system. Hogan (1984) analyzed the role of joint stiffening caused by cocontraction of an antagonistic setup for maintaining joint position (when no external torque is applied) in a simulation study in comparison to active control, asking, when do we need cocontraction and when does an actively controlled reflexive stiffness suffice? Similarly, Akazawa et al. (1983) investigated changes in stretch reflex gain and stiffness of the long thumb flexor muscles in a force-control and a constant-load position control task. Gribble et al. (2003) explored the relationship between cocontraction and the target size in a pointing task. Osu et al. (2002) investigated short- and long-term changes in cocontraction when interacting in known and unknown environments. Selen et al. (2005) analyzed in a simulation study whether cocontraction leads to more joint stability or larger fluctuations in the paradoxical situation that both stability and motor noise increase with muscle activation. Grebenstein et al. (2011) hypothesized about criteria for joint stiffening by observing examples from sports.

Cocontraction increases the stiffness of arm joints, at least in the absence of external forces (Osu et al., 2002). It is a successful strategy to stably maintain a position when internal models of the environment are imprecise, when external perturbations are expected but not predictable, or when perturbation frequencies are too high for the central nervous system to react (Akazawa et al., 1983; Hogan, 1984; Osu et al., 2002). Cocontraction can also be a successful strategy for decreasing trajectory variability and improving endpoint accuracy during multijoint arm movements (Gribble et al., 2003). The ability of cocontraction to stabilize a limb “…highly depends on levels of motor noise and sources, and on muscular architecture and skeletal properties…” (Selen et al., 2005).

Cocontraction probably also plays an important role for the absorption of impact energy (Grebenstein et al., 2011). In case of known impact energy, humans adapt joint stiffness to dissipate the impact energy over a broad range of joint motion inside the joint limits to avoid damage to the muscles. For unknown impacts, humans use a strategy of maximum cocontraction to dissipate as much energy as possible using their muscles knowing that reaching joint limits causes substantially more irreversible injuries.

However, the influence of cocontracting extrinsic and intrinsic antagonistic pairs of hand muscles on decoupling grip stiffness from its usual increase with grip force remains an open question. The investigation of the effect of cocontraction on stiffness is rather limited, and existing studies investigated the usage of cocontraction at *zero net force* only, i.e., no forces are applied to the environment. The usage of forces is highly relevant for interacting with the environment and the manipulation of objects and possibly the ability to alter stiffness at this force, too.

From VSAs in robotics, we know about their limited ability to decouple stiffness and torque for the higher torques. Is this true for human locomotor system, as well? Is the ability of decoupling force and stiffness using cocontraction limited to the lower force ranges, e.g., to zero net force, since intrinsic stiffness increases with force anyway? Or are we able to considerably decouple the two also for the higher forces? To address this question, this study will focus on human’s ability to decouple stiffness from its linear increase with force using cocontraction.

Two ways of forcing subjects to cocontract are acknowledged, either by (a) the application of unstable force fields (Akazawa et al., 1983) or by (b) presenting a visual feedback about the applied muscular activity from relevant muscle groups (Osu and Gomi, 1999; Osu et al., 2002; Shin et al., 2009). Using unstable force fields seems to force subjects to increase cocontraction in a natural way but is probably limited to the production of zero net force, which means that no forces are applied by the finger or limb. On the other hand, forcing subjects to produce cocontraction based on measured *electromyography* (EMG) is an unnatural task, but allows to command different combinations of contraction and cocontraction including those leading to non-zero net force. However, so far it has been used only to investigate different levels of cocontraction at zero net force.

In this study, we will use a completely different approach (c) and present visual feedback of the applied force and stiffness of each *prior* trial to a participant, allowing him or her to learn how to modulate stiffness over the course of multiple trials.

## 2 Materials and Methods

We measured stiffness in subject experiments with and without voluntary cocontraction using a device that applies a fast position perturbation to a thumb–index finger grip. We used optical tracking to observe and prevent changes in kinematics and electromyography to analyze and investigate the regression of force and stiffness from muscular activity.

### 2.1 Device Description

**Figure 2:**
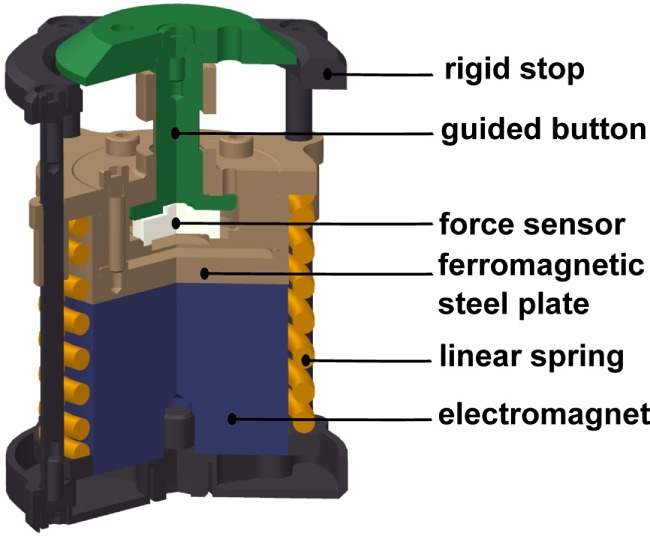
**Cross-sectional view of the grip perturbator**.

The grip perturbator we used in this experiment is presented in Figure 2. A spring (orange) is preloaded by an electromagnet (blue) fixed to a frame (black) that holds a moving part (brown). The grip force is measured with a load cell (white). Releasing the spring causes the device to elongate by 7.5 mm within a few milliseconds (see perturbation force profile in Figure 5). Amendments since our previous study (Höppner et al., 2013) concern an improved guiding of the gripping force to the small load cell and allows for a smaller grip length. In addition, three markers for optical tracking and two small fans were attached to reduce the heating caused by the electromagnet. The perturbator weighs 165 g, and its length varies between 54 and 61.5 mm. The spring force is 140 N when loaded and 100 N when unloaded, i.e., considerably higher than the pinch grip force, ensuring identical experimental conditions independent of how firmly the perturbator is held. The load cell is a KM10 (ME-Messsysteme GmbH) force sensor with a nominal sensitivity of 1 mV/V and a nominal range of 100 N. The accuracy of the analog signal provided by the measurement amplifier GSV-11H (ME-Messsysteme GmbH) is 0.1 N.

#### 2.1.1 Electromyography

Keeping in mind a possible application in telerobotics, we use non-invasive surface electrodes rather than invasive needle electrodes. The surface electrodes *Delsys Trigno Wireless System* have an internal amplification of 1 kV/V and provide an analog signal at 4 kHz with a constant delay of 48 ms. These electrodes complies with the requirements put forth by the Medical Device Directive 93/42/EEC, and we comply with its intended use. The EMG electrodes were attached in accordance with the recommendations of the SENIAM project (Hermens et al., 2000). Before the experiment, the subjects were asked to wash their arm with water; no soap was used. For an optimal EMG signal, the respective part of the skin was again moistened with water. As a result of earlier prestudies, we have chosen in total six muscles to be relevant for our experimental procedure: two extrinsic index flexor muscles (FDP and FDS), two extrinsic index extensor muscles (EIP and ED), and two interossei muscles in the hand (FDI and SDI; see Table 1). Please note that even if SDI inserts at the middle finger, we found a strong influence on our measurements and thus decided to include it.

**Table 1:** **Investigated muscles and their function (Schünke et al., 2005)**.

Muscle	Abbreviation	Function
M. flexor digitorum superficialis	FDS	Wrist flexion; flexion of the metacarpophalangeal and the proximal interphalangeal joints of index, middle, ring, and little finger
M. flexor digitorum profundus	FDP	Wrist flexion; flexion of the metacarpophalangeal, the proximal interphalangeal, and the distal interphalangeal joints of index, middle, ring, and little finger
M. extensor digitorum	ED	Extension of the metacarpophalangeal, the proximal interphalangeal, and the distal interphalangeal joints of index, middle, ring, and little finger
M. extensor indicis proprius	EIP	Extension of the metacarpophalangeal, proximal interphalangeal, and distal interphalangeal joints of the index finger
Mm. interossei dorsales I/II	FDI/SDI	Flexion of the metacarpophalangeal joints of the index and middle finger; extension and abduction of the proximal and the distal interphalangeal joints of the index and middle finger

Within the earlier prestudies, which were conducted without any tests for significance and thus not published, we analyzed in a force task the influence of index finger stiffness only. We found similar stiffness values and force–stiffness relations as measured in a pinch grasp. Since we found the index finger predominating the measured grip stiffness, we concluded the thumb to be much stiffer than the index finger. Thus, within this study, we refrained from measuring EMG of corresponding muscles of the thumb (flexor pollicis longus, extensor pollicis brevis, and extensor pollicis longus).

Furthermore, we tested measuring the adductor pollicis muscle as well. Due to strong sweating and large movement of the underlying skin for the pinch grip, the electrodes took off very rapidly, which makes it impossible for us to measure this muscle. The electrodes were placed close to the six corresponding muscles (see Figure 4) by the subjects using palpation and visual feedback of the EMG signal.

#### 2.1.2 Optical Tracking

The positions of arm and fingers were continuously monitored through optical tracking and corrected where necessary, so as to prevent variations from kinematics. The optical tracking system is a *Vicon Motion Capture System* consisting of 8 *MX3+* cameras and an *MX Ultranet* controller. The cameras were arranged at distances between 0.5 and 1 m around the forearm position (for all subjects the same). The cameras have an optimal resolution of 659 (horizontal) × 494 (vertical) pixels at 242 frames per second, and we used them at a frequency of 400 Hz. After positioning the EMG sensors, marker sets for tracking the position and orientation of wrist and forearm and single markers to track the positions of the distal phalanx of index finger and thumb were positioned (see Figure 4). The optical tracking system was calibrated using the orientation of the table. The idea of the optical tracking system was to give the subject and the experimenter a feedback about variations in kinematics during the experiment to constrain it and correct when necessary, rather than using the measured optical tracking data to identify influences and their significance. We decided to use optical tracking rather than different cuffs to constrain the kinematics since it offers more possibilities for the subjects to choose a relaxed initial posture and avoids occupying suitable EMG positions. Furthermore, there is no risk that the subjects apply wrist torque against the cuff, the influence of which on the EMG signal we would not be able to quantify.

#### 2.1.3 Graphical User Interface

**Figure 3:**
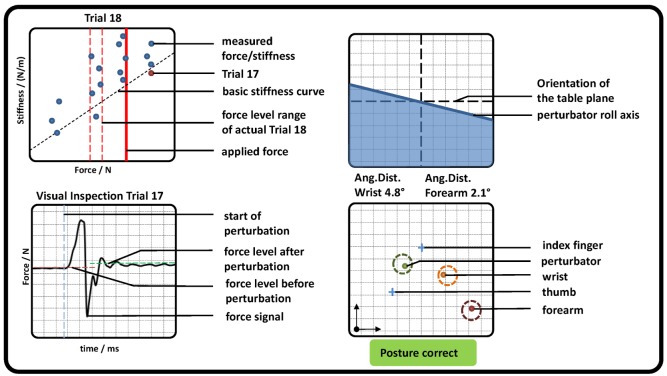
**Graphical representation of measured pose and force data, which were presented to the subjects (representative)**. (Top left) Applied force (red solid line) and goal force level (red dashed lines). All previously measured perturbations were depicted as blue dots showing the applied force and stiffness, while the very last was highlighted in red. The estimation of the basic stiffness curve achieved in task 1 was depicted as a diagonal black dashed line. (Bottom left) The last perturbation was depicted for visual inspection for artifacts. Furthermore, the detected mean forces before and after perturbation as well as its beginning were shown. (Top right) The roll axis of the perturbator and its radial deflection in reference to the table plane (similar to an attitude indicator in an airplane). (Bottom right) The position of perturbator, index, thumb, wrist, and forearm depicted as dots in a plane parallel to the table. In addition, a circle with a radius of 15 mm was plotted, which indicates a tolerance around each initial measured position. If all dots were inside each circle, a text “Posture correct” was shown in green; otherwise a comment “CAUTION!! Correct posture!” was shown in red.

In addition, subjects saw a graphical representation of the measured data on a screen (see Figure 3). For controlling the force, two red dashed lines and one red solid line representing the required force level and the measured force were depicted. Directly after each perturbation, the measured stiffness and force were visually presented to the subject as a dot in a force–stiffness graph. This procedure allows the subject and the experimenter to check the subject’s performance in the preceding trial. Furthermore, the following kinematic information was presented to the subjects: the planar positions of forearm, wrist, perturbator, thumb, and index finger; the orientation of the longitudinal perturbator axis (roll axis) in reference to the table plane; and the angular distances of wrist and forearm in reference to their initial orientations. The subjects were asked to keep the positions of the perturbator, the wrist, and the forearm within tolerance ranges, depicted as circles with a radius of 15 mm around the initial captured positions. They were furthermore asked to keep the orientations of the wrist and the forearm (displayed as angular distances in Figure 3) close to the initially detected ones and the roll axis of the perturbator parallel to the table plane. Note that for a successful perturbation, the force was controlled automatically to be kept within a certain force range; despite that, the positions were just visually inspected by the experimenter and not constrained to avoid fast fatigue of the subjects. As soon as the release button for valid perturbation conditions was pressed by the experimenter, the perturbation was applied after a random interval between 0.5 and 2.5 s.

**Figure 4:**
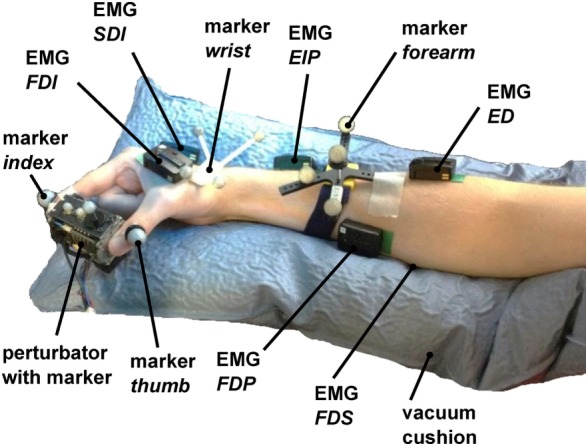
**Measurement setup**. The perturbator was held by the subject between index finger and thumb, while middle finger, ring finger, and pinky had to be flexed. 6 EMG electrodes were placed to corresponding flexor and extensor muscles on the hand (FDI and SDI) and forearm (FDP, FDS, EIP, and ED). The forearm was placed in a vacuum cushion to assist subjects with holding their wrist and arm position stable. The positions of index finger, thumb, perturbator, wrist, and forearm and the orientations of perturbator, wrist, and forearm were tracked with an optical tracking system.

The measurement setup consisted of a host computer running Linux, a real-time target computer running QNX, and a Windows computer. The real-time computer runs a MATLAB/Simulink model to control the electromagnet, to read out the force sensor at 10 kHz, and to read out the EMG sensors. The marker positions were recorded with the Windows computer and transferred to the Linux host using the DLR communication protocol *arDNet* (Bäuml and Hirzinger, 2008). A triggered recording of the Vicon data was started at 250 ms before each perturbation and lasted for 1 s. Measured force signals were calibrated before each trial since the output of the force sensor was marginally influenced by the heating of the electromagnet.

### 2.2 Experimental Procedure

A total of 10 healthy subjects, nine male and one female (S3), seven right and three left-handed (S5, S7, S9), age 22–27 years, and all initially fully naive to the experiment, performed the two experimental protocols, with and without isometric cocontraction, as described below. For all subjects and experiments, the right hand was used, be they right or left handed, which is restricted by the design of the perturbator with its fans and optical markers. To further assist the subjects in holding their wrist and arm orientation stable during the measurements, a vacuum cushion was used, which was adjusted to each subject. Subjects were seated in all experimental conditions.

The whole procedure lasted between 90 and 120 min per participant. No subject had a history of neurological disorder or neuromuscular injury affecting the CNS or the muscles. All subjects participated voluntarily and gave written consent to the procedures, which were conducted in partial accordance with the principles of the Helsinki agreement (non-conformity concerns the point B-16 of the 59th World Medical Association Declaration of Helsinki, Seoul, October 2008: no physician supervised the experiments). Approval was received from the works council of the German Aerospace Center, as well as its institutional board for data privacy ASDA; the collection and processing of experimental data were approved by both committees.

At first, subjects were asked to lay their arm relaxed on the table to measure the *EMG base noise* level for 5 s (see Appendix). Furthermore, the initial poses of wrist, forearm, and perturbator and the positions of index finger and thumb were measured in this relaxed pose. Second, subjects were asked to fulfill *maximum voluntary contraction* (MVC), i.e., to grip as strongly as they were able to, three times for 5 s each, while the maximum grip force and corresponding EMG levels were measured. The MVC was used to set the prescribed force levels in the following two main tasks.

#### 2.2.1 Task 1—Force Task without Voluntary Cocontraction

In *task 1*, subjects were asked to stably hold six different visually presented force levels using the vertical red lines (15, 25, 35, 45, 55, and 65% of MVC) within a range of ±5% of MVC without using any kind of voluntary cocontraction. The force levels were given to them in a randomized order four times each, leading to a total of 24 perturbations. The perturbation is a small and fast displacement of 7.5 mm of the pinch grip, and force is measured to calculate stiffness using its difference before and after perturbation. Since active response is not our scope, the measurement is finished within 40 ms. This procedure is similar to the one in our previous studies (Höppner et al., 2011, 2013), except that wrist and finger positions were measured and constrained, and EMG was measured. This force task is considered to deliver information about the subject’s basic stiffness and its dependency on force.

A linear fit between force and stiffness was calculated from the measured perturbations and plotted as the basic stiffness curve in the force–stiffness graph (black dashed line in Figure 3 top left).

#### 2.2.2 Task 2—Force Task with Isometric Cocontraction

In *task 2*, subjects were asked to produce a force using the red vertical lines and to further decouple stiffness from force by using isometric cocontraction. Before *task 2*, subjects had the possibility to learn how to increase grip stiffness voluntarily by cocontraction using 10 to 20 trials that were not recorded. After this learning procedure, subjects were asked to reach 5 different force levels (15, 25, 35, 45, and 55% of MVC) given to them in a randomized order within a range of ±5% of MVC 15 times each and use cocontraction to produce higher stiffness at a similar force than in *task 1*, leading to 75 perturbations. In other words, they had to keep the red solid line between the two red dashed lines and always produce stiffness higher than the black dashed line in Figure 3. After each set of 25 perturbations, the subjects paused for 5 min. During these breaks, again the EMG base noise was recorded for 5 s to detect strong deviations. After all perturbations, the subjects were asked to produce three times the MVC level for 5 s again. Note that this method does not allow commanding certain cocontraction levels. It is unfeasible to require subjects reaching a force–stiffness combination twice and can be probably only achieved after days of learning, if possible at all. This method only allows commanding the force, and the cocontraction level depends on the subject’s effort.

### 2.3 Data Processing

From the measured force data and the known position perturbation, we calculated the grip stiffness. We found out from the optical tracking data how the perturbation length is distributed to thumb and index finger. We evaluated whether and how well stiffness and force values could be predicted from EMG data and how EMG–force and EMG–stiffness relationships vary within and across subjects. We analyzed whether and how much voluntary cocontraction and the grip force before the perturbation influenced stiffness, EMG values, and kinematics.

#### 2.3.1 Determination of Force and Stiffness

**Figure 5:**
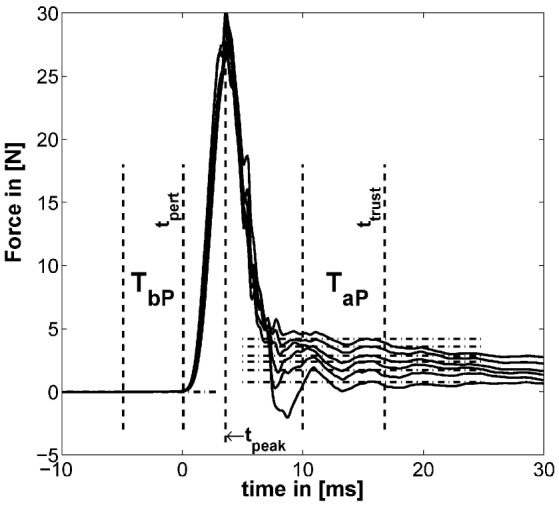
**Example for typical perturbation profile of a performed force task without cocontraction**. Force profile before, during, and after perturbation starting at *t* = 0. In addition, the time windows *T*_bP_ and *T*_aP_ and the mean of force for six force levels are depicted (mean force $E_{{\text{T}}_{\text{bP}}}\left(f\right)$ subtracted). The length of *T*_aP_ and *t*_trust_ were found to be optimal at 18.33 and 33.3 ms, respectively [adapted from the study by Höppner et al. (2013)].

The methods to define the two time windows *T*_bP_ before and *T*_aP_ after the perturbation are similar to the one introduced in our previous study (Höppner et al., 2013) [see Figure 5 adapted from the study by Höppner et al. (2013)], which is performed offline.

The force signals *f* were first filtered using a 21-point moving average filter. We defined the start of the perturbation *t*_pert_ as the end of the first time interval *T*_bP_ lasting 10 ms. *T*_bP_ is the last time interval before *t*_peak_ (the peak after the perturbation/maximum of the force signal), which has a standard deviation (SD) below 5 ⋅ 10^−4^ N. This number was empirically determined and led to stable results. The force before the perturbation was calculated using *T*_bP_. Assuming that neuromuscular feedback does not have any measurable influence within 40 ms (Höppner et al., 2013), the time *t*_trust_, which starts after perturbation and within which one can ignore effects of fast reflex responses, was allowed to vary between *t*_pert_ ≤ *t*_trust_ ≤ *t*_pert_ + 40 ms and the duration *T*_aP_ between 5 and 20 ms so as to minimize the objective function

(1) $$Z=\frac{1}{n_{\text{sub}}}\sum_{i=1}^{n_{\text{sub}}}\left(\frac{1}{n_{\text{level}}}\sum_{j=1}^{n_{\text{level}}}\left(\tilde{e}\left(k_{{\text{task}1}_{\mathrm{ij}}}\right)+\frac{1}{n_{\text{trial}}}\sum_{k=1}^{n_{\text{trial}}}\left({\tilde{e}}_{T_{\text{aP}}}\left(f_{{\text{task}1}_{\mathrm{ijk}}}\right)+{\tilde{e}}_{T_{\text{aP}}}\left(f_{{\text{task}2}_{\mathrm{ijk}}}\right)\right)\right)\right)$$

using all trials *n*_trial_, levels *n*_level_, and subjects *n*_sub_. The operator $\tilde{e}\left(\cdot \right)\geq 0$ denotes the *coefficient of standard error* we introduced recently (Höppner et al., 2013), which combines the coefficient of variation and the standard error (SE), and which has no unit. The SE compensates the SD *σ*(⋅) for sample size *n* assessing low sample sizes with a higher SE; the coefficient of variation is a normalized measure of the SD and compensates for the sample mean μ(⋅). Since the objective function equation (1) mixes data sets of different size (force and stiffness) and from different dimensions (time window length and number of repetitions), we had to compensate the SD *σ*(⋅) for both. The minimum of this cost function minimizes the variation of resulting stiffness values *k* measured under *exactly* the same conditions (which is true for *task 1*, only) and the oscillations in force within time interval *T*_aP_ of both tasks. Since subjects cannot produce the exact same cocontraction level twice (see section Experimental Procedure), and thus, the experimental conditions between perturbations in *task 2* cannot be trusted to be identical, the part of the objective function that accounts for variations in measured stiffness considers *task 1*, only. The stiffness *k* of each trial was calculated using

(2) $$k=\frac{E_{T_{\text{aP}}}\left(f\right)-E_{T_{\text{bP}}}\left(f\right)}{x_{\text{aP}}-x_{\text{bP}}},$$

where $E_{{\text{T}}_{\text{bP}}}\left(\cdot \right)$ and $E_{{\text{T}}_{\text{aP}}}\left(\cdot \right)$ denote the average over time intervals *T*_bP_ and *T*_aP_ before and after perturbation. Note that the displacement *x*_aP_ − *x*_bP_ was for all experimental conditions constant (see section Device Description). The length of the second time interval *T*_aP_ and its end *t*_trust_ were found to be optimal under named constraints at 18.3 and 33.3 ms, respectively.

For investigating intrasubject and intersubject variability, force and stiffness were normalized subjectwise by their maximum values and divided by their SDs.

The influence of both tasks on the stiffness was analyzed statistically, as explained in the paragraph *Methods for Testing Statistical Significance* below.

#### 2.3.2 Evaluation of Optical Tracking Data

Since the optical tracking data were sometimes subject to artifacts, we detected the beginning of the perturbation within these data for each trial manually and synchronized the data sets from the real time and windows machine manually. For determining finger and thumb displacement caused by the perturbation, we applied the same time windows as for estimating stiffness from force. Furthermore, the measurements of the single markers at the index finger and thumb were not stable and sometimes flipped. Thus, we implemented a procedure that allocates these two markers according to their distance from the perturbator.

In addition, these two marker positions sometimes switched for a few milliseconds to unreasonably high values or to exact zero, which we detected automatically and discarded as missing information. For evaluating the kinematics, we used two main metrics, the SD of the distance to describe the variation in position and, if available, the SD in angular distance to describe the variation in orientation (see section Appendix). While the distance was calculated using the Euclidean norm, we calculated the angular distance between two rotation matrices *R*_1_ and *R*_2_ according to the study by Stillfried et al. (2014):

(3) $$\text{angdist}:=\text{arccos}\left(\frac{\text{trace}\left({\text{R}}_{2}\cdot {{\text{R}}_{1}}^{-1}\right)-1}{2}\right).$$

Since the kinematic position was controlled to be kept stable and not commanded *per se*, we refrained from analyzing the influence of kinematics on stiffness and from drawing wrong conclusions. Thus, its remaining influence is still part of the measurement noise.

#### 2.3.3 Processing of the EMG Data

The oversampled EMG signal (analog card sampling inside the real-time target computer rate 10 kHz; sampling rate of the EMG signal provided by the Delsys Trigno Wireless EMG system 4 kHz) was filtered offline using a delay-free second-order Butterworth bandpass filter between 25 and 450 Hz. The produced muscular activity was evaluated using the average rectified value (ARV) over a time frame of 200 ms before the perturbation. From the relaxation task, a steady time window of about 500 ms was chosen manually (identical for all electrodes within a task), representing the EMG base noise level. The base noise of each electrode was subtracted from the EMG data subjectwise. EMG data were normalized by their maximum values and divided by their SDs for each electrode and each subject.

#### 2.3.4 Regression of Force and Stiffness from EMG and Evaluation of Its Intrasubject and Intersubject Variability

We built regression models of force and stiffness from EMG using $f_{i}=\beta_{1}+\bar{\beta }\cdot \bar{\text{EMG}}$ and $k_{i}=\beta_{1}+\bar{\beta }\cdot \bar{\text{EMG}}+\beta_{n}\cdot f_{i}$. A clear focus is set on intersubject regression, since it allows for a subject-independent measurement of force and stiffness from muscular activity for teleoperating compliance of modern robotic hands. We divided all force and stiffness data of each subject by their SDs, since they are expected to vary considerably between subjects. The regressed models are cross-validated; for intrasubject regression, we predicted each trial subjectwise by building a model regressed from all other trials (leave-one-trial-out; see section Appendix), while for intersubject regression, we predicted all trials of one subject with a model regressed from all other subjects (leave-one-subject-out). As a measure of each model fitness, the cross-validated coefficient of determination *R*^2^ was used. For calculating the intrasubject *R*^2^ cross-validated values the number of required models equals the number of perturbations per subject (leave-one-trial-out) and for the intersubject *R*^2^ cross-validated values the number of required models equals the number of subjects (leave-one-subject-out) were used. Since we expected a non-linear dependency between measured EMG and force, we tested if taking the square root (Hogan, 1984) or square (Shin et al., 2009) of all EMG data improves the quality of the linear fits in force and stiffness.

#### 2.3.5 Methods for Testing Statistical Significance

For significance testing, we first performed a multivariate two-way repeated-measure MANOVA to reveal whether there are significant influences of the factors *task* and *force level* and their interaction on the obtained dependent variables stiffness, kinematics, and EMG values. For the single dependent variables, we performed a univariate two-way repeated-measure ANOVA with a *post hoc* Tukey’s honestly significant difference (THSD) test to reveal significant patterns of the two factors. Moreover, for testing significance of a correlation, we used a standard function in MATLAB, which provides a *p* value based on results of a *t*-test testing differences in variances. Equality of variances was tested using a two-sample F-test. Finally, Steiger’s z-test was used to investigate differences between correlations (Steiger, 1980).

## 3 Results

**Figure 6:**
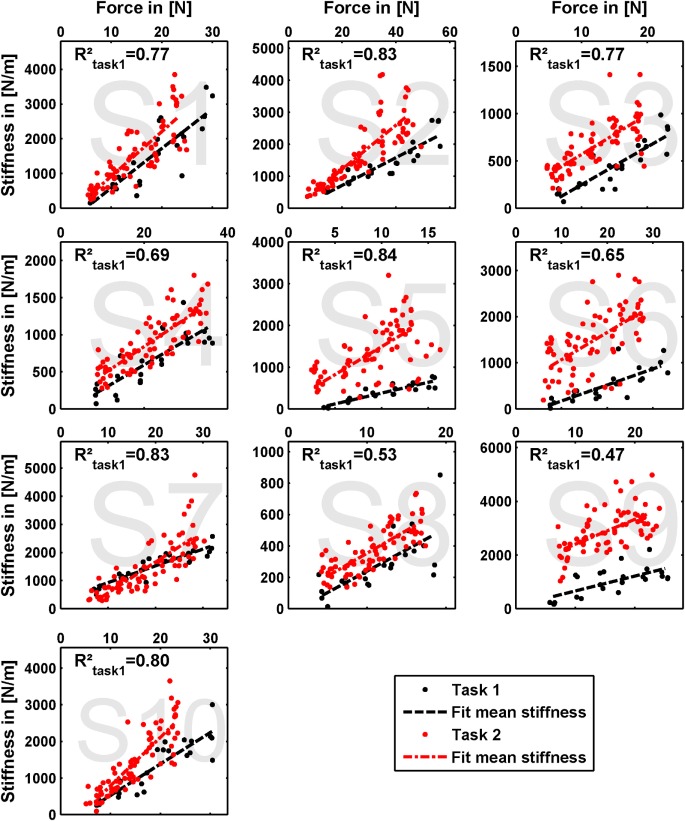
**Measured grip stiffness and its dependency on grip force**. The results are depicted as dots denoting the single perturbations. For both tasks, a linear regression between force and stiffness over all values is shown. For *task 1*, we additionally calculated the corresponding coefficient of determination ${\text{R}}_{\text{task}1}^{\text{2}}$ as a measure of linearity.

The results of our measurements are shown as force–stiffness plots in Figure 6. The results are depicted as dots denoting the single perturbations. For both tasks, a linear regression between force and stiffness over all values is shown. For *task 1*, we additionally calculated the corresponding coefficient of determination $R_{\text{task1}}^{2}$ as a measure of linearity.

**Figure 7:**
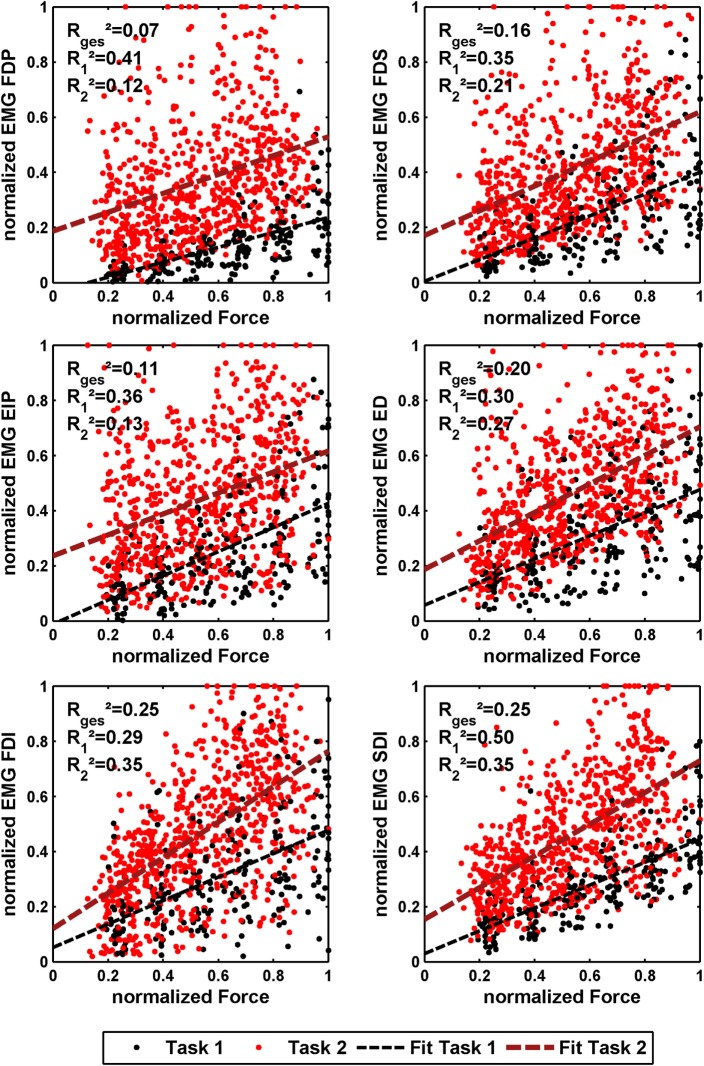
**Normalized force depending on normalized EMG of all 10 subjects for the 6 different EMG electrodes**. The black dots denote the results of *task 1*, and the red ones denote the results of *task 2*. In addition, a linear regression is depicted for both. The coefficient of determination is given for a linear fit of each single task and both tasks together.

The effect of force production and voluntary cocontraction on the normalized electromyogram of each of the six electrodes is depicted in Figure 7.

We performed a multivariate two-way repeated-measure MANOVA—including the dependent variables *stiffness*, *EMG*, *thumb*, and *index finger* displacements—to reveal whether there was a significant influence of the factors *task* and *force level*. The results showed that both factors (*p* ≤ 0.001) and their interaction (*p* ≤ 0.05) have a significant influence on the obtained results.

Concerning effects of learning and fatigue, we found no significant correlation between trial number to both force and stiffness for the experimental condition of *task 1*. There is a significant positive correlation for subject S6 between trial number and stiffness and a significant negative correlation for subject S5 between trial number and force for the experimental condition of *task 2*.

### 3.1 Ability to Cocontract and Decouple Stiffness from Force

The linear regressions in Figure 7 show the expected increase of activations across all electrodes from *task 1* to *task 2*. Results of Figure 6 reveals clearly the expected influence of voluntary cocontraction on stiffness. Performing a univariate two-way repeated-measure ANOVA for the dependent variable *stiffness* showed that both factors *task* and *force level* (*p* ≤ 0.01) are significant, but their interaction is not significant. *Post hoc* THSD tests revealed a significantly larger stiffness within *task 2* and—as might be expected—an always increasing stiffness with *force level* (*p* ≤ 0.0001). Moreover, two measures for the ability to *decouple stiffness from force* are given in Table 2 for the different force levels over the pooled trials of all subjects. The stiffness values are normalized per subject by their maximum value. The baseline stiffness at each force level is given in the first and third row as the mean of stiffness in *task 1*, $\left\langle k_{{\mathrm{task}1}_{i}}^{*}\right\rangle$, and its SD $s\left(k_{{\mathrm{task}1}_{i}}^{*}\right)$, in which subjects are asked to produce simply force without cocontraction. In the second and forth row, the mean stiffness of *task 2*, $\left\langle k_{{\mathrm{task}2}_{i}}^{*}\right\rangle$, and its SD $s\left(k_{{\mathrm{task}2}_{i}}^{*}\right)$ are given, in which the subjects try to increase stiffness by cocontraction. The difference $\left\langle k_{{\mathrm{task}2}_{i}}^{*}-k_{{\mathrm{task}1}_{i}}^{*}\right\rangle$ and their ratio $\left\langle k_{{\mathrm{task}2}_{i}}^{*}/k_{{\mathrm{task}1}_{i}}^{*}\right\rangle$ in the fifth and sixth row are two different measures exhibiting the average voluntary increase in stiffness through cocontraction.

**Table 2:** **Mean difference and ratio between normalized stiffnesses of the two tasks for the single force levels**.

	10% MVC	20% MVC	30% MVC	40% MVC	50% MVC
$\left\langle {\text{k}}_{\text{task}{\text{1}}_{i}}^{*}\right\rangle$	8.7%	18%	22%	35%	40%
$\left\langle {\text{k}}_{\text{task}{\text{2}}_{i}}^{*}\right\rangle$	23%	32%	42%	56%	66%
$\text{s}\left({\text{k}}_{\text{task}{\text{1}}_{i}}^{*}\right)$	±5.7%	±7.7%	±8.7%	±16%	±15%
$\text{s}\left({\text{k}}_{\text{task}{\text{2}}_{i}}^{*}\right)$	±12%	±14%	±16%	±17%	±16%
$\left\langle {\text{k}}_{{\mathrm{task}\text{2}}_{i}}^{*}-{\text{k}}_{{\mathrm{task}\text{1}}_{i}}^{*}\right\rangle$	15%	14%	21%	21%	26%
$\left\langle {\text{k}}_{\text{task}{\text{2}}_{i}}^{*}/{\text{k}}_{\text{task}{\text{1}}_{i}}^{*}\right\rangle$	5.3	2.2	2.2	1.9	1.9

*The mean values in stiffness $\left\langle {\text{k}}_{\text{task}{\text{1}}_{\text{i}}}^{*}\right\rangle$ and $\left\langle {\text{k}}_{\text{task}{\text{2}}_{\text{i}}}^{*}\right\rangle$ of the two tasks and their SDs $\text{s}\left({\text{k}}_{\text{task}{\text{1}}_{\text{i}}}^{*}\right)$ and $\text{s}\left({\text{k}}_{\text{task}{\text{2}}_{\text{i}}}^{*}\right)$ are given. In addition, mean difference in normalized stiffness $\left\langle {\text{k}}_{\text{task}{\text{2}}_{\text{i}}}^{*}-{\text{k}}_{\text{task}{\text{1}}_{\text{i}}}^{*}\right\rangle$ and their ratio $\left\langle {\text{k}}_{\text{task}{\text{2}}_{\text{i}}}^{*}/{\text{k}}_{\text{task}{\text{1}}_{\text{i}}}^{*}\right\rangle$ for all force levels in percent of MVC over all subjects are listed. Note that the index *i* denotes the mean over subjects*.

### 3.2 Kinematics

Beside minimizing the variation in kinematic orientation and position during the experiments, the kinematic data reveal insights on how the total perturbation length of 7.5 mm is distributed between thumb and index finger and give an indication of the relative stiffnesses of the two digits. Table 3 provides the results of the finger and thumb perturbation displacements for all subjects with respect to the wrist frame, their average values, and SDs in percent; all displacements are divided by the total perturbator displacement of 7.5 mm (2.5% of the data is zero and thus deleted; see section Data Processing). Note that we related the thumb and index finger position before and after perturbation to the wrist frame instead to the world coordinate frame to get rid of forearm movements interpreted as grip displacements; anyway, both lead to similar results (world coordinate frame related data not listed).

**Table 3:** **Perturbation displacement of index finger and thumb**.

Subjects	S1	S2	S3	S4	S5	S6	S7	S8	S9	S10	Mean ±SD
Index $\bar{{\text{x}}_{{\text{T}}_{\text{b/aP}}}}$ [%]	67	63	65	85	67	56	82	63	77	71	69
s [%]	±7.7	±8.5	±22	±13	±7.4	±10	±8.7	±14	±13	±13	±15
Thumb $\bar{{\text{x}}_{{\text{T}}_{\text{b/aP}}}}$ [%]	31	33	35	28	24	38	23	29	31	28	30
s [%]	±4.3	±8.9	±5.8	±8.0	±4.0	±8.3	±6.3	±11	±15	±7.3	±9.7
Total $\bar{{\text{x}}_{{\text{T}}_{\text{b/aP}}}}$ [%]	98	96	100	112	91	94	104	93	108	98	100
s [%]	±9.9	±4.9	±21	±10	±5.3	±13	±10	±6.6	±23	±12	±15

*Mean and SD *s* of index finger and thumb displacements $x_{{\text{T}}_{\text{b}/\text{aP}}}$ between before and after perturbation in [%]. The displacements are divided by the total perturbator displacement of 7.5 mm. 2.5% of the data set was deleted*.

Performing univariate two-way repeated-measure ANOVAs for the dependent variables *thumb* and *index finger* displacements showed that the factor *force level* is significant for both variables (*p* ≤ 0.05), but the factor *task* is significant for the index finger displacement (*p* ≤ 0.01), only. *Post hoc* THSD tests revealed no significant pattern for both factors and variables.

For details about how subjects performed in keeping the predefined position, please have a look into the Appendix.

### 3.3 Regressing Force and Stiffness from EMG

We performed an intersubject regression of stiffness and force from EMG (see Tables 4 and 5). The results showed a large influence of the muscular activity of FDI, SDI, and force to the regression of stiffness, while all electrodes except EIP contributed equally to the regression of force. The coefficient of determination of both models highly differs between both regressions across all subjects: 72 ± 12% and 33 ± 18% for regressing stiffness and force, respectively. The mean correlation coefficients and their SDs between stiffness, force, and muscular activity across all subjects are listed in Table 6. By using these values, we conducted a paired *t*-test on the Fisher-transformed correlation coefficients on whether the correlation of EMG to force and stiffness significantly differs across subjects. The results show that only for the two intrinsic muscles in the hand, the correlation of EMG to stiffness significantly differs in comparison to its correlation to force (*p* ≤ 0.001). A detailed analysis of the correlations between force, stiffness, and muscular activities for each of the two tasks can be found in the Appendix. Moreover, an overview on the contributions from the three groups of muscles—extrinsic extensors and flexors and interossei—can be found here.

**Table 4:** **Intersubject regression of stiffness from force and EMG**.

Subjects	S1	S2	S3	S4	S5	S6	S7	S8	S9	S10
EMG_FDP_	+++	–	–	++	.	–	–	–	–	–
EMG_FDS_	–	–	+	–	.	++	.	++	+	+
EMG_EIP_	–	–	–	++	–	–	–	.	–	–
EMG_ED_	–	–	–	–	–	–	–	–	+	.
EMG_FDI_	+++	+++	+++	+++	+++	+++	+++	+++	+++	+++
EMG_SDI_	+++	+++	+++	+++	+++	+++	+++	+++	+++	+++
Force	+++	+++	+++	+++	+++	+++	+++	+++	+++	+++
R^2^ [%]	61	90	67	70	79	62	90	57	68	76

*–, no significance; ., *p* ≤ 0.05; +, *p* ≤ 0.01; ++, *p* ≤ 0.001; +++, *p* ≤ 0.0001*.

*The significance of the respective coefficients and models’ coefficient of determination are listed for each subject. Note that the model is cross-validated (leave-one-subject-out)*.

**Table 5:** **Intersubject regression of force from EMG**.

Subjects	S1	S2	S3	S4	S5	S6	S7	S8	S9	S10
EMG_FDP_	+	++	+++	+	+++	+++	+++	+++	+++	+++
EMG_FDS_	++	++	+++	–	+++	+++	+++	+++	+++	+++
EMG_EIP_	.	–	–	–	–	–	–	–	–	–
EMG_ED_	+++	++	+++	+++	+++	++	+++	+++	+++	+++
EMG_FDI_	+	++	+	+	+++	+++	+++	++	+++	++
EMG_SDI_	+++	+++	+++	+++	+++	+++	+++	+++	+++	+++
R^2^ [%]	15	55	9	14	26	38	47	53	24	49

*–, no significance; ., *p* ≤ 0.05; +, *p* ≤ 0.01; ++, *p* ≤ 0.001; +++, *p* ≤ 0.0001*.

*The significance of the respective coefficients and models’ coefficient of determination are listed for each subject. Note that the model is cross-validated (leave-one-subject-out)*.

**Table 6:** **Correlation between stiffness, force, and EMG**.

r [ ]	Stiffness	Force
EMG_FDP_	0.53 ± 0.25	0.32 ± 0.31
EMG_FDS_	0.55 ± 0.20	0.45 ± 0.33
EMG_EIP_	0.48 ± 0.30	0.38 ± 0.38
EMG_ED_	0.57 ± 0.25	0.52 ± 0.35
EMG_FDI_	0.81 ± 0.10	0.57 ± 0.14
EMG_SDI_	0.76 ± 0.10	0.53 ± 0.11
Force	0.65 ± 0.19	–

*Mean and SD of correlation coefficients between stiffness, force, and muscular activity across subjects and tasks. By using these values, we conducted a paired *t*-test on the Fisher-transformed correlation coefficients on whether the correlation of EMG to force and stiffness significantly differs across subjects. The results show that only for the two intrinsic muscles FDI and SDI, the correlation of EMG to stiffness significantly differs in comparison to its correlation to force (*p* ≤ 0.001)*.

Since literature inconsistently reports, we tested whether taking the square root or square of EMG data improves the quality of the linear fits of force and stiffness to EMG using Steiger’s z-test (Steiger, 1980). The tests showed that the plain muscular activity provides a better correlation to both force (*p* < 0.001) and stiffness (*p* < 0.05) than taking the square of muscular activation. Moreover, no clear improvement can be found by taking the square root in comparison with plain muscular activity. Finally, taking the square root of muscular activity in comparison to the square clearly improves its correlation to force (*p* < 0.01), but not to stiffness. Conclusively, all reported results and analyses focusing on regressing stiffness and force from EMG use the plain muscular activity.

For details about intrasubject regression, please have a look into the Appendix.

## 4 Discussion and Conclusion

In this article, we analyzed the role of voluntary cocontraction for decoupling grip stiffness from its natural increase with grip force. To measure influences from cocontraction only, we minimized effects of variabilities in kinematics by providing the subject a visual feedback of the current hand and arm posture. In a first task, we asked subjects to apply a set of force levels several times without the use of cocontraction to measure the basic force–stiffness coupling. In a second task, we asked subjects to decouple stiffness from force using voluntary cocontraction while holding a specific force level. We measured EMG to investigate the possibility of regressing stiffness and force from the measurement of muscular activity.

### 4.1 Ability to Decouple Stiffness from Force by Cocontraction

The results show that the subjects were able to increase grip stiffness between 15 and 26% of maximum stiffness by the use of cocontraction. By using the difference $\left\langle k_{{\mathrm{task}2}_{i}}^{*}-k_{{\mathrm{task}1}_{i}}^{*}\right\rangle$, the results show an increasing ability with force (*r* = 0.30, *p* < 0.05). Milner and Franklin reported in the study by Burdet et al. (2013) based on results of Milner (2002) a 5-fold range in modulation of wrist stiffness at zero net joint torque. Similarly, subjects in our experiment were able to modulate stiffness by cocontraction in a 5.2-fold range for the lowest force level. On average, subjects were able to vary stiffness $\left\langle k_{{\mathrm{task}2}_{i}}^{*}/k_{{\mathrm{task}1}_{i}}^{*}\right\rangle$ with cocontraction by a 2.7 ± 2.2-fold range (maximum at first force level of subject S5 with a 22-fold and minimum at second force level of subject S7 with a 0.8-fold modulation in stiffness).

The results provide an overview to what extent the human is able to decouple grip stiffness from force using cocontraction, while probably revealing only parts of it: First, subjects in our study had problems to stably hold the lower force levels at high cocontraction, where effects of motor noise on hand shaking are considerably higher (which confirms the supposition that cocontraction is the wrong strategy to stably hold a force level). Similarly, Kearney and Hunter (1990) reported in a study performed at the human ankle that subjects had difficulties achieving cocontractions involving high levels of muscle activations at zero net torque. Thus, subjects in our experiments probably did not use their full ability to decouple stiffness from force for the lower force levels, while they did for the higher ones. Maybe the strategy we used in our experiments of restricting subjects to exactly hold a force level is not the optimal solution for the lower levels. A better strategy might be monitoring the steadiness of force as a perturbation criterion, while the experimenter supervises the force range to help subjects reaching the higher cocontraction levels for the lower forces. Nevertheless, there is evidence suggesting that neural mechanisms of muscle inhibition and excitation exist, which limit the ability to produce all possible sets of cocontractions, probably to avoid harming the muscular system (De Luca and Mambrito, 1987). On the other hand, Milner (2002) reported that subjects were not able to voluntarily apply maximal cocontraction, but could possibly increase it by days of training similar to the study by Darainy et al. (2004). Furthermore, *task 2* in our experiments was performed up to forces of 55% MVC, only. As we found in our study (Höppner et al., 2011), this constraint avoids fast fatigue of corresponding muscles for subjects during this long-lasting experimental procedure, but does not allow us to draw conclusions about forces up to 100% MVC.

To have similar cocontraction ranges at all force levels, we commanded in a former version of the experiments a combination of applied force and EMG similar to the work done by Osu and Gomi (1999), Osu et al. (2002), and Shin et al., 2009. We merged the different EMG signals into one lumped signal and asked subjects to hold different combinations of force and summarized EMG; so instead of commanding stiffness, we commanded an EMG level, which should be related to cocontraction in some way. Due to the high density of muscles in the forearm lying in different layers and thus high cross talk of multiple muscles, subjects learned to produce the EMG levels and simultaneously learned to reduce the metabolic costs for producing it. This resulted in subjects successfully solving the task without producing an increase in the measured grip stiffness. This led to the decision for a redesign of the experiments and to command grip stiffness *per se* rather than a combined EMG level.

Anyway, similar to our results reported above, Akazawa et al. (1983) found that the reflex responsiveness and stretch-evoked stiffness increase linearly with cocontraction as defined in their article. Also, the slope of this increase is steeper, the larger the tonic force is, corresponding to our result of an increasing stiffness modulation capability with higher force. However, please note that Akazawa et al. (1983) only compared the cocontraction levels of two tonic force levels achieved in the constant-load position control task and measured reflex-affected stiffness.

Finally, it needs to be mentioned that our finding of an increasing ability for decoupling force and stiffness by cocontraction is opposing the torque–stiffness plots of existing VSA mechanisms (as mentioned in section Introduction), which have a rather limited ability to decouple stiffness from torque, especially for the higher torques. The force–stiffness plots we measured within this study allow for the first time for a suitable insight and can be helpful information for robotic engineers designing VSAs.

### 4.2 Finger Displacement

The evaluation of tracked kinematics show that for all experimental conditions the index finger got perturbed by about 2/3 and the thumb by about 1/3 of the whole displacement (see Table 3). This means that *the thumb is approximately twice as stiff as the index finger*. Assuming that both, the measured intrinsic stiffness and the force correspond to the number of attached crossbridges [(Burdet et al., 2013), p. 41f.], this means that *the thumb is also approximately twice as strong as the index finger*. This theory is backed by the findings of Olafsdottir et al. (2005), who showed MVC finger forces of thumb and index of 73 ± 18 and 33 ± 6.6 N, respectively. During their measurements, all digits were activated simultaneously and the thumb opposed the other fingers. Nevertheless, it remains unclear whether this ratio is dominated by stiffer muscles or a difference in moment arms of index finger and thumb in a pinch grip.

### 4.3 Regressing Stiffness and Force from EMG

We built for each subject a linear model using all other subjects and used it to estimate the stiffness/force data based on muscular activity and force (leave-one-subject-out cross-validation). Even if the subject is unknown, these models provide surprisingly good results for the regression of stiffness. However, this holds for the regression of stiffness, only, and not for force. What is the reason? The significances of the coefficients for these two regressions show that the two intrinsic muscles in the hand had an unexpectedly high influence on the modeling of stiffness, while all muscles contributed almost equally to the regression of force. Looking into correlations between stiffness, force, and muscular activities shows a comparatively high correlation of the intrinsic muscles to stiffness (see Table 6). Moreover, the SDs of these correlations are significantly less for the two intrinsic muscles than for the extrinsic ones (*p* < 0.05) meaning that these muscles provide a stable correlation across subjects. This is possibly a result of higher signal-to-noise ratio (SNR) for the intrinsic muscles. Since the measured surface EMG signal involves the EMG pattern from other, deep, muscles—which we interpret as a lower SNR for the extrinsic muscles—the correlation of forearm muscles dropped, while the one of the intrinsic muscles in the hand did not. Similarly, Maier and Hepp-Reymond (1995) reported for almost all intrinsic hand muscles about “…high correlations to grip force with low variability, whereas the majority of the extrinsic muscles, with the exception of the long flexors, have lower correlations and higher individual variability…” in an isometric pushing task.^1^ Conclusively, the possibility for a suitable regression of stiffness as it is influenced by voluntary cocontraction across subjects is caused by a high and stable correlation between stiffness and intrinsic muscular EMG across all subjects.

But can we conclude from these differences for regressing force and stiffness that the interossei predominate the decoupling of stiffness, perhaps by having a steeper increase of stiffness with force, while force is produced by all groups of muscles equally? Or is it just the case that the intrinsic muscles are simultaneously activated with muscles that we do not measure with EMG, but which contribute to the measured stiffness?

First, we need to acknowledge that prestudies led us to the wrong conclusion of a predominant role of the index finger on the measured stiffness, based on which we decided to exclude muscles activating the thumb from the EMG measurements. But since we find the thumb to be *just* twice as stiff, we cannot reason a dominating role of the index finger with certainty. Thus, we cannot clarify plausibly if it is causality (intrinsic muscles predominate cocontraction) or just correlation (intrinsic muscles are synergistically activated) from the conducted experiments. But the result can be interpreted from a biomechanical point of view: coactivating extrinsic flexor and extensor muscles introduces high forces on the finger joints. This may lead to instability at—in particular—the metacarpophalangeal joint: it could reduce the strain by an uncontrolled sideways, abduction-like, movement. The interossei muscles, connecting the proximal and metacarpal bones at each side of the metacarpophalangeal joint, can be used to stabilize this movement—and apparently do. A somewhat similar mechanism can be found, e.g., during pinch grip: extrinsic extensor muscles—namely extensor carpi ulnaris and extensor carpi radialis longus/brevis—are activated simultaneously with flexor muscles to prevent the wrist from moving; i.e., the intent is to contract the flexor muscles, and the extensor muscles are activated involuntarily to provide support.

Moreover, a publication from Milner et al. (1995) argues in an opposite way: from an investigation of moment arms and physiological cross-sectional areas of the first dorsal interosseus and lumbricalis muscles, they revealed that these muscles must have a predominant role for controlling the force direction at the index finger, while the extrinsic muscles in the forearm act as stabilizers. Hence, they concluded that extrinsic muscles should contribute much more to finger stiffness.

It needs to be acknowledged that the SDI does neither control index finger nor thumb and controls the movement of the middle finger, only (see Table 1). However, due to a high influence we measured in prestudies, we decided to include this electrode. The performed experiments prove this initial finding with a large influence of the gathered SDI activity on stiffness. This is possibly caused by either a synergistic activation of this muscle or the measurement of cross talk from other muscles, e.g., first palmar interosseus.

Note that we investigated the use of non-linear regression models, as well, to improve the results: Gaussian processes (Rasmussen and Williams, 2006), linear regression with random Fourier features (Rahimi and Recht, 2007), and neural networks. None of these methods showed a significant improvement of model fitness over the linear approach, which is why we neglect them in this study. We hypothesize that the small amount of data available (approximately 100 data points for 10 subjects) does not allow to fully leverage the power of more expressive models.

On the basis of the results in the studies by Joyce and Rack (1969) and Vrendenbregt and Rau (1973), Hogan (1984) reported a linear dependency between muscle force and measured EMG activation until 30% of maximum voluntary contraction and a muscle force proportional to the square root of the pooled firing rate. Contrary, Shin et al. (2009) proposed that muscle tension follows a quadratic function of measured activation. Thus, we tested whether applying a square or square root to our processed EMG data would improve the fit. The results show that taking the square root or square of muscular activity neither improves its correlation to force nor improves its correlation to stiffness. Moreover, the results show that taking the square even makes the correlations worse. However, our measurements include levels of 55% of MVC only and do not allow us to draw conclusions for the higher force levels.

All in all, the intrinsic muscles in the hand are found to dominate our regression of stiffness and not of force, while the experiment design does not allow us to reveal whether the stiffness itself is dominated by these muscles. A good possibility to answer this question might be the use of functional electrical stimulation placed on respective extrinsic and intrinsic muscles as performed for the human hand (Lauer et al., 1999) or for the intrinsic plantar foot muscles in the study by Kelly et al. (2014), which was not the focus of the experiments performed in this study. Nevertheless, the result is promising: the high SNR and high correlation to stiffness of the intrinsic hand muscles allow for a continuous measurement of grip stiffness and to explain on average 72 ± 12% of its variance without any prior knowledge about the subject, i.e., calibration of stiffness to force and EMG in advance. This information allows to continuously teleoperate finger stiffness to actively impedance controlled robotic hands, as well as hands based on VSAs (Grebenstein et al., 2011). Moreover, it allows to continuously measure a *task-dependent stiffness* during activities of daily living: Leidner et al. (2015) started categorizing *Compliant Manipulation Tasks* into a task taxonomy, e.g., by classifying tasks of contact/no contact, in-hand manipulation/external manipulation tasks, or tasks with and without deformation of the environment. By continuously measuring stiffness of the hand, it will be possible to measure a *task dependent stiffness* during activities of daily living, such as cutting an onion, cleaning with a sponge or connecting a plug (Leidner et al., 2015), and to add a meaningful range of stiffness values to the derived taxonomy matrix.

## Author Contributions

HH developed the idea and device, researched the literature, analyzed the data, and contributed to the acquisition of data. MG-D contributed to acquisition of data and the analysis of data. GS contributed to the acquisition and analysis of data and revised the work. JB contributed to the regression analysis and testing of machine-learning methods. PS contributed to the interpretation of data and revised the work.

## Conflict of Interest Statement

The authors declare that the research was conducted in the absence of any commercial or financial relationships that could be construed as a potential conflict of interest.

## Figures and Tables

**Figure A1:**
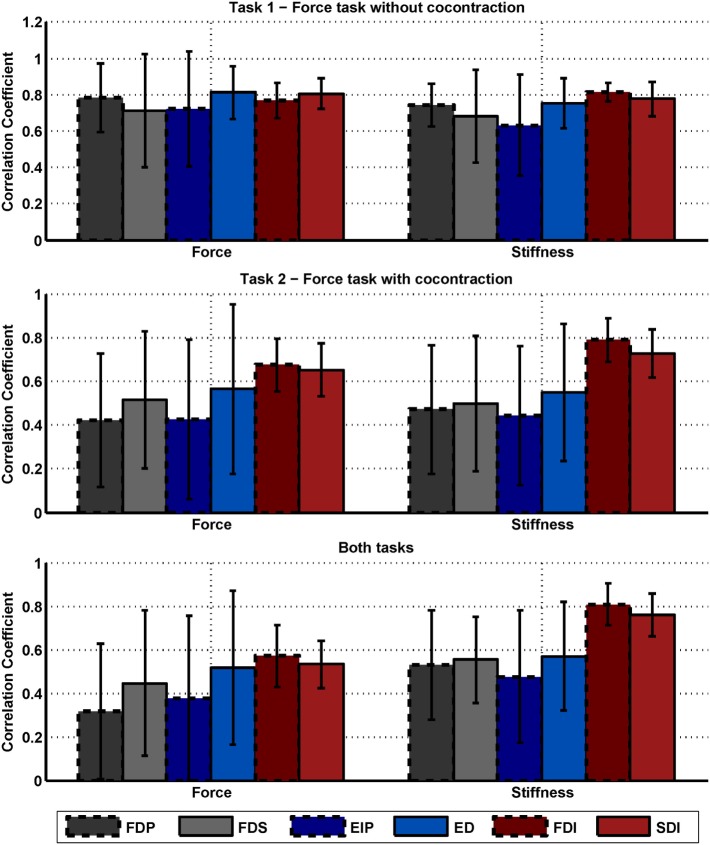
**Correlation between force, stiffness, and muscle activations**. Mean correlation coefficients between force, stiffness, and the single muscle activations and their SDs across subjects for the two tasks. The SDs across 10 subjects are depicted as error bars. The diagrams on the top, middle, and below show the results of *task 1*, *task 2*, and for both tasks, respectively. Note that the results of the diagram are redundant to the information provided in Table 6.

**Figure A2:**
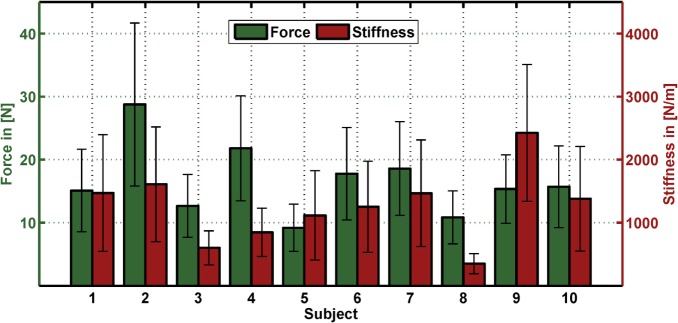
**Mean and SD in force and stiffness for each subject**. The bar depicts the mean and the error bar the corresponding SD for both force and stiffness for each subject across all values.

**Figure A3:**
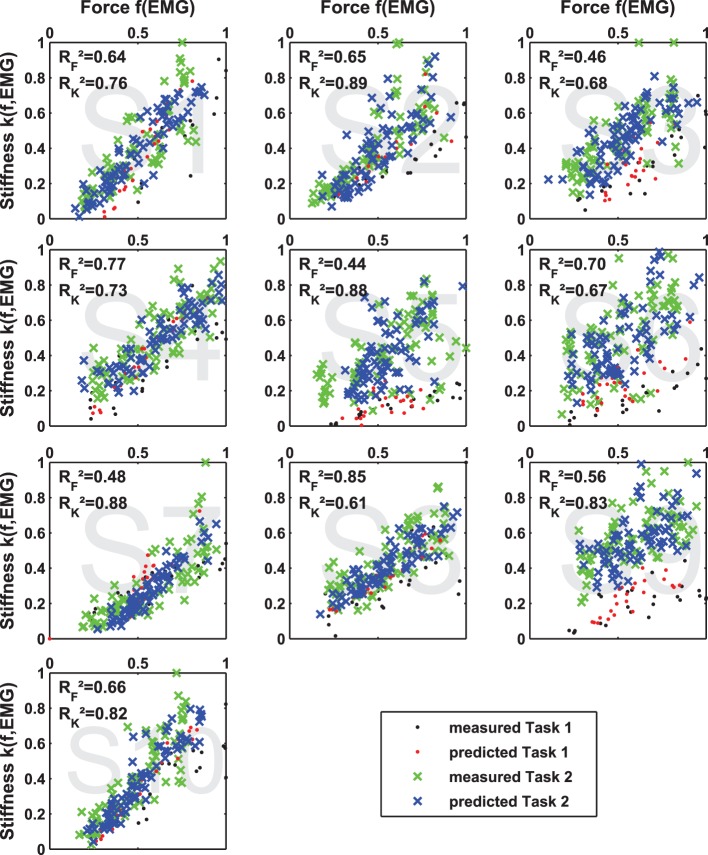
**Independence of predicted data of intrasubject regression**. Results of multiple linear regression of stiffness *k*(*f*, EMG) and force *f* (EMG) and their coefficients of determination *R*^2^ in comparison to the measured values for both tasks. If the predicted values are located more or less on a line, the two regression models are most likely not linear independent, and the content of information of the respective EMG signals reduces to one.

**Figure A4:**
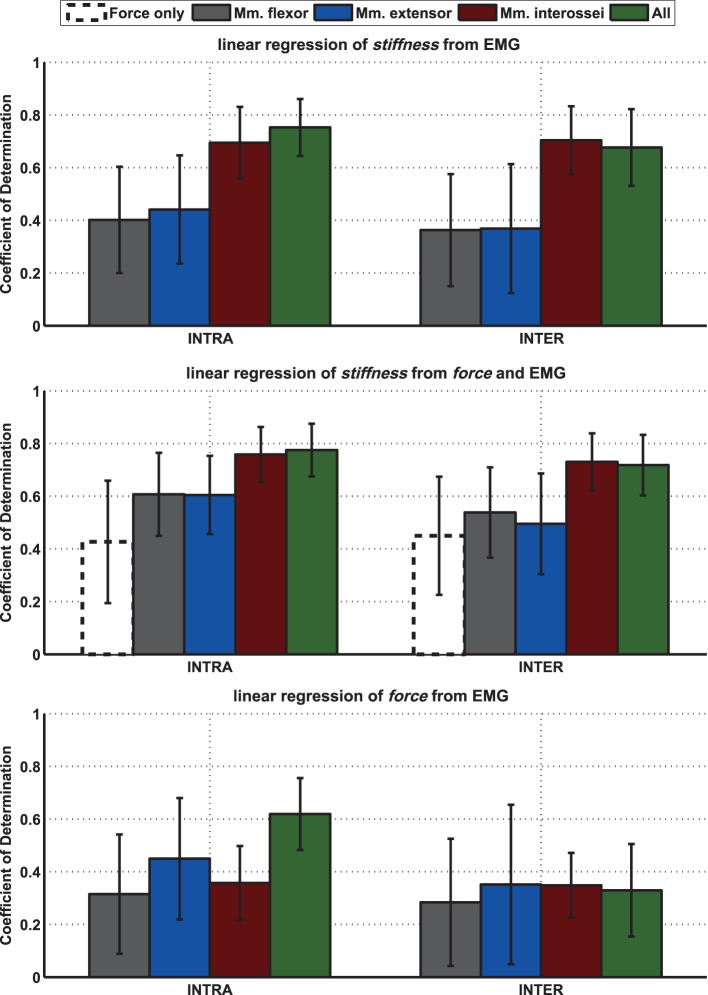
**Mean of intrasubject and intersubject coefficients of determination R^2^ for different linear models between stiffness, force, and EMG and results of performed statistical testing**. Mean leave-one-out cross-validated coefficient of determination *R*^2^ for intrasubject (left; leave-one-trial-out) and intersubject (right; leave-one-subject-out) stiffness and force over all subjects for regressing stiffness from EMG or force, from EMG and force, and for regressing force from EMG using different muscle groups. The SDs over 10 subjects are depicted as error bars.

## Footnotes

^1^Please note that the authors of Maier and Hepp-Reymond (1995) asked subjects for the production of low isometric forces, only, and not for voluntary cocontraction. Similarly, we reported an overview of correlation coefficients between force, stiffness, and muscular activities for both tasks in the Appendix.

## Appendix

### A Kinematics

Table A1 in Appendix lists the variation in distance of all markers over all subjects in reference to the world coordinate frame and in reference to each other (0.7% of the optical tracking data is zero and thus deleted). Table A2 in Appendix does the same for the orientation of perturbator, wrist, and forearm (0.6% of the optical tracking data is zero and thus deleted). The marker position and orientation of the forearm of subject S8 was controlled during the experiment but not recorded for some unknown reason. The SD of the horizontal orientation of the perturbator is found to be ±2.95 (see Figure 3).

**Table A1:** **SDs s in distance between all tracked markers**.

s [mm]	Thumb	Index	Pert.	Wrist	Forearm	World
Thumb	–	±0.72	±0.72	±1.7	±3.8	±3.9
Index	±0.72	–	±1.2	±1.4	±2.9	±2.8
Pert.	±0.72	±1.2	–	±2.3	±3.4	±3.4
Wrist	±1.7	±1.4	±2.3	–	±2.3	±2.7
Forearm	±3.8	±2.9	±3.4	±2.3	–	±2.2
World	±3.9	±2.8	±3.4	±2.7	±2.2	–

*SDs in distance over all subjects for the single tracked markers index finger, thumb, perturbator, wrist, and forearm inside *T*_bP_ in [mm] in reference to each other and to the world coordinate system. 0.7% of the data set was deleted*.

**Table A2:** **SDs s in angular distance between all tracked markers**.

s [^∘^]	Pert.	Wrist	Forearm	World
Pert.	–	±3.5	±3.0	±3.3
Wrist	±3.5	–	±3.2	±3.2
Forearm	±3.0	±3.3	–	±1.4
World	±3.3	±3.2	±1.4	–

*SDs in angular distance over all subjects for the single tracked markers perturbator, wrist, and forearm inside *T*_bP_ in [^∘^] in reference to each other and to the world coordinate system. 0.6% of the data set was deleted*.

The displacement of the index finger is found to be slightly decreasing (*test statistics for correlation r* = − 0.17; *p* ≤ 0.001) and the displacement of the thumb slightly increasing (*test statistics for correlation r* = 0.20; *p* ≤ 0.001) with force over all subjects, while there is no significant correlation to stiffness. Furthermore, there is a slight increase of index finger and thumb displacement (*test statistics for correlation r* = 0.16 and *r* = 0.077; *p* ≤ 0.025) with the number of perturbations (duration of the experiment).

### B EMG Base Noise

In the relaxing task, a mean base noise ARV of 5.8 ± 1.7 μV over all subjects and electrodes was measured, which is consistent with literature (Konrad, 2005).

### C Correlations between Force, Stiffness, and Muscular Activities for Both Tasks

Figure A1 in Appendix shows the mean correlation coefficients between force, stiffness, and the single muscle activations and their SDs across subjects for the two tasks. Maier and Hepp-Reymond (1995) reported for almost all intrinsic hand muscles about “…high correlations to grip force with low variability, whereas the majority of the extrinsic muscles, with the exception of the long flexors, have lower correlations and higher interindividual variability…” high correlations in an isometric pushing task. We can confirm a good correlation to force in a pure pushing task. Moreover, the low intersubject variability for the intrinsic muscles in comparison to the extrinsic is obvious as well in all our experimental conditions for both force and stiffness.

Differences regarding mean values between the two tasks and between correlations to force and stiffness are clearly visible. While the correlations are similar for the condition of the isometric pushing *task 1*, they differ for the condition of *task 2* and the usage of voluntary cocontraction. Moreover, the strong correlation of the intrinsic muscles to stiffness (but not force) across both experimental conditions can be seen.

### D Regressing Stiffness and Force from EMG

For regressing force and stiffness from the measured muscular activity, we normalized the force and stiffness values and divided them by their SD subjectwise (see section Data Processing). Figure A2 in Appendix shows for each subject the mean and SD across all values for both force and stiffness.

Tables A3 and A4 in Appendix list the results of an intrasubject regression of stiffness and force from EMG (leave-one-trial-out cross-validation). Similar to the results of the intersubject regression, a dominant role of FDI and SDI can be seen for the regression of stiffness, while all muscles contribute equally to the regression of force. Thereby, the coefficient of determinations are 78 ± 10% and 62 ± 14% for regressing stiffness and force, respectively. Naturally, the intrasubject regression provides a better fit in comparison to the intersubject regression. Again, the regression of stiffness from muscular activity performs better than the regression of force.

**Table A3:** **Intrasubject regression of stiffness from force and EMG**.

Subjects	S1	S2	S3	S4	S5	S6	S7	S8	S9	S10
EMG_FDP_	+++	–	.	–	+++	–	+	–	++	–
EMG_FDS_	+	–	–	–	–	–	–	–	++	–
EMG_EIP_	–	–	.	–	–	–	.	–	++	.
EMG_ED_	–	–	–	–	–	–	–	.	+	+
EMG_FDI_	–	+++	+	++	+++	+++	+++	–	+++	+
EMG_SDI_	.	+++	–	–	++	+	++	+	++	–
Force	+++	+++	+++	+	–	–	+++	–	–	+
R^2^ [%]	76	89	68	73	88	67	89	61	83	83

*–, no significance; ., *p* ≤ 0.05; +, *p* ≤ 0.01; ++, *p* ≤ 0.001; +++, *p* ≤ 0.0001*.

*The significance of the respective coefficients and models’ coefficient of determination are listed for each subject. Note that for calculating the coefficients of determination the model is cross-validated (leave-one-trial-out)*.

**Table A4:** **Intrasubject regression of force from EMG**.

Subjects	S1	S2	S3	S4	S5	S6	S7	S8	S9	S10
EMG_FDP_	+++	+++	–	++	–	–	–	–	+	.
EMG_FDS_	++	–	+	+++	+++	.	–	++	–	+
EMG_EIP_	–	–	+++	+++	+	+	+	+	+++	+
EMG_ED_	–	+++	+	+++	+	+++	–	+++	+++	++
EMG_FDI_	+	–	++	++	–	–	–	++	–	.
EMG_SDI_	.	++	.	–	–	–	–	–	–	–
R^2^ [%]	64	65	46	77	44	70	47	85	56	66

*–, no significance; ., *p* ≤ 0.05; +, *p* ≤ 0.01; ++, *p* ≤ 0.001; +++, *p* ≤ 0.0001*.

*The significance of the respective coefficients and models’ coefficient of determination are listed for each subject. Note that for calculating the coefficients of determination the model is cross-validated (leave-one-trial-out)*.

Moreover, Figure A3 in Appendix shows plots of measured and predicted stiffness and force data using intrasubject regression for both tasks. These plots show how much of the independence of force and stiffness can be extracted from the EMG signals. If the predicted force–stiffness points cover the same area as the measured force–stiffness points, their independence is completely retained after the prediction from EMG. If the predicted points lie on a line, their independence is completely lost and the information content of the EMG signal is reduced to one.

### E Contributions of Muscle Groups to the Regression of Force and Stiffness

Besides the influence from each single muscle and electrode, it is of interest how the muscle groups—extrinsic flexors, extrinsic extensors, and intrinsic interossei, with two electrodes each—contribute to the regression of stiffness and force from EMG. Moreover, it is of interest how much the information of force adds to the regression of stiffness. The results of a linear regression on intrasubject and intersubject variability of stiffness and force from EMG (and force) are plotted in Figure A4 in Appendix. As a measure of each model fitness, the cross-validated coefficient of determination *R*^2^ is used. For calculating the intrasubject *R*^2^ cross-validated values the number of required models equals the number of perturbations per subject (leave-one-trial-out) and for the intersubject *R*^2^ cross-validated values the number of required models equals the number of subjects (leave-one-subject-out). This analysis provide 4 interesting results: (a) Using muscular activity of the intrinsic muscles in the hand to regress stiffness provides a considerably better fit than using EMG of any extrinsic muscle group, which is true for both intrasubject and intersubject regression. (b) Using the intrinsic muscle, EMG works considerably better than just using force for the regression of stiffness. What’s more, it seems that adding additional state information, namely, the measured grip force, does not add much to the regression of stiffness as it is decoupled from force. (c) Similar to the analysis of correlation coefficients in Figure A1 in Appendix, the **SD** of the intrinsic muscles to regress stiffness across subjects is comparably low, which is why these muscles allow for a suitable intersubject regression, as well. (d) The regression of force from EMG works totally differently, i.e., there is no dominating role of the intrinsic muscles. If at all, the extrinsic extensors seem to dominate here. But for an adequate intrasubject regression of force from EMG, the information of all muscles is necessary. While the regression of stiffness is found to be working for intersubject regressions as well, an intersubject regression of force from EMG is not.
